# The Effect of Calcium Ions on Resting Membrane Potential

**DOI:** 10.3390/biology13090750

**Published:** 2024-09-23

**Authors:** Elizabeth R. Elliott, Robin L. Cooper

**Affiliations:** Department of Biology, University of Kentucky, Lexington, KY 40506, USA; erel222@uky.edu

**Keywords:** calcium, ion substitution, K2P channels, membrane potential, potassium

## Abstract

**Simple Summary:**

The factors that influence electrical potential across the cell membrane are important because they affect cellular properties that, in turn, allow tissues, organs, and whole organism to function; as such, it is important to understand them. The distribution and movement of certain ions (i.e., Na^+^, K^+^, Ca^2+^) determine this electrical potential as they move across the membrane through channels and transport processes, some of which are impeded by the presence of other ions. As a particularly apt example: free Ca^2+^ ions are known to block open Na^+^ channels. This study illustrated that raising the Ca^2+^ ion concentration on one side of the membrane led to a larger membrane potential, and this is suggested herein to be caused by blockage of a leaky Na^+^ channel. If more such channels are present in the membrane, the effects of altering Ca^2+^ concentration have less impact.

**Abstract:**

Regulating membrane potential is key to cellular function. For many animal cells, resting membrane potential is predominantly driven by a family of K2P (two-pore domain) potassium channels. These channels are commonly referred to as leak channels, as their presence results in the membrane being permeable to K^+^ ions. These channels, along with various pumps and exchangers, keep the cell resting membrane potential (Rp) relatively close to potassium’s equilibrium potential (E_K_); however, in many cells, the resting membrane potential is more depolarized than the E_K_ due to a small Na^+^ ion leak. Raising [Ca^2+^]_O_ (extracellular Ca^2+^ concentration) can result in hyperpolarization of the membrane potential from the resting state. The mechanism for this hyperpolarization likely lies in the blockage of a Na^+^ leak channel (NALCN) and/or voltage-gated Na^+^ channels. The effects may also be connected to calcium-activated potassium channels. Using *Drosophila melanogaster*, we here illustrate that changing [Ca^2+^]_O_ from 0.5 to 3 mM hyperpolarizes the muscle. Replacing NaCl with LiCl or choline chloride still led to hyperpolarization when increasing [Ca^2+^]_O_. Replacing CaCl_2_ with BaCl_2_ results in depolarization. K2P channel overexpression in the larval muscle greatly reduces the effects of [Ca^2+^]_O_ on cell membrane potential, likely because potential is heavily driven by the E_K_ in these muscles. These experiments provide an understanding of the mechanisms behind neuronal hypo-excitability during hypercalcemia, as well as the effects of altered expression of K2P channels on membrane potential.

## 1. Introduction

Resting membrane potential (Rp) is known to vary amongst cells, both within an organism and even at the tissue level, which has been attributed to differences in the types of ion channels present and the density of the channels themselves [1]. Some cells utilize ligand-gated channels or those moderated by cellular responses, but the channels primarily involved in the electrical activity for conduction are passive or voltage-gated channels [2]. The specific distribution of these channels varies by cell, as each has a unique molecular composition. However, at rest, the two main channels present are the two pore-domain potassium (K2P) and sodium (NALCN) leak channels [2,3]. Since most cells have a larger P_K_ (permeability to K^+^ ions) than P_Na_ (permeability to Na^+^ ions), the cell’s Rp is largely driven towards the potassium equilibrium potential (E_K_).

Interestingly, variations in extracellular Ca^2+^ ([Ca^2+^]_O_) are known to result in altered Rp without Ca^2+^ flux [4]. [Ca^2+^]_O_ is known to affect conduction in central neurons and play a large role in synaptic transmission [5,6,7]; it has also been proposed that Ca^2+^ affects Rp through the activation of calcium-activated potassium channels (K_Ca_) and/or alteration in the kinetics or blockage of voltage-gated Na^+^ channels [8,9] and NALCN channels [2,3]. The Rp of a cell is generally more depolarized than the E_K_ value but raised [Ca^2+^]_O_ can block NALCN channels and cause hyperpolarization [3]. The NALCN channel was biophysically characterized by Lu et al. [10] and the importance of its role in physiology and pathological conditions is discussed in Monteil et al. [3]. Ca^2+^ ions block some types of voltage-gated ion channel, particularly of the Na^+^ variety [8,9,10,11,12], but the resulting hyperpolarization can also potentially remove the residual inactivation of voltage-gated Na^+^ channels and thus lower the threshold of a cell’s excitability. Changes to electrical potential may also occur as Ca^2+^ alters the screening of charges across membranes [13].

This investigation—regarding the influence of Ca^2+^ ions on membrane potential—was conducted with the muscles of larval *Drosophila melanogaster*. The effects of ion substitution were observed through a reduction [Na^+^] of the bathing saline via replacement of the NaCl with choline chloride or LiCl. Ion substitution replacing Ca^2+^ with Ba^2+^ was conducted to address whether similar hyperpolarization is observed during exposure to another divalent cation capable of conduction through Ca^2+^ channels, as well as to investigate whether some small amount of Ca^2+^ might leak into the cell when [Ca^2+^]_O_ is high, since Ba^2+^ would not activate the K_Ca_ channels present [14]. Various buffers were used and brought to an appropriate pH with HCl to maintain physiological levels while avoiding the use of NaOH or NaHCO_3_.

Using *Drosophila* as a model allowed for genetic alteration of channel expression (specifically, that of K2P channel subtypes) and adjustment of the relative relationship between P_Na_ and P_K_, which helped determine whether high [Ca^2+^]_O_ conditions still illustrate a reduction of P_Na_ through NALCN channels. Altered K2P expression is known to occur (to varied degree depending on the channel subtype involved) in cancerous/diseased mammalian tissues, though it is yet unknown whether they are a cause or a consequence of these pathologies [15,16]. Hypo- and hypercalcemia are known to have physiological effects on the excitability of human neurons, with the former possibly resulting in Chvostek’s sign (twitches of the face due to activation of the motor nerve) or Trousseau’s sign (slow contraction of the hand muscles), though the specifics of these effects, other health outcomes, and even the mechanisms behind altered [Ca^2+^] _O_ on membrane excitability are not fully understood [17,18].

Knowing that larval *Drosophila* muscle has an E_Cl_ of around −40 mV, [19,20] an E_K_ of about −90 mV [21,22], and [Ca^2+^]_O_ of around 1.5 mM in the hemolymph [23] provides a foundational understanding of physiological parameters that would be helpful while trying to address alterations in membrane function caused by changes in [Ca^2+^]_O_. Additionally, the fact that *Drosophila* represent a genetically amenable model allows examination of how altered expression of K2P channels affects Rp and the effects of varied [Ca^2+^]_O_ on it. This study is intended to provide an initial assessment of how altered [Ca^2+^]_O_ affects the Rp of *Drosophila* muscle to foster future studies into the detailed mechanism behind the observations included herein. *Drosophila* genetics being easily manipulable renders them a good model for determining how altered expression of specific proteins affects regulation, allowing the support or refutation of proposed mechanisms.

## 2. Materials and Methods

### 2.1. Animals

*Drosophila melanogaster* Canton S (CS) flies were used in physiological assays. This strain was originally obtained from the Bloomington *Drosophila* Stock Center (BDSC) but has been isogenic in the laboratory since 1996. *Drosophila* CS larvae were used as early third instars (50–70 h post-hatching). Overexpression of the ORK1 protein in larval body wall muscles (m6 and m7) was achieved by crossing homozygous males of BG487 (BDSC stock # 51634) with female virgins of UAS-ORK1 (BDSC stock # 6586). Progeny carrying one copy each of GAL4 driver and UAS-ORK1, referred to as body muscle m6-m7 > ORK1, were used. Larval body wall muscles 6/7 feature an anteroposterior gradient pattern of BG487-Gal4 expression, allowing BG487 to drive UAS–ORK1 in those muscles [24,25]. The larvae were maintained at room temperature, ~21 °C, in vials partially filled with a cornmeal-agar-dextrose-yeast medium.

### 2.2. Dissection and Physiology

Similar dissection procedures and electrophysiological measures have been described previously [26]. Briefly, transmembrane potentials were monitored in m6 muscles of early third-instar larvae using sharp intracellular electrodes (30 to 40 megaOhm resistance) filled with 3 M KCl. An Axonclamp 2B (Molecular Devices, Sunnyvale, CA, USA) amplifier and 1 X LU head stage was used. LabChart 7.0 (ADInstruments, Colorado Springs, CO, USA) was used to collect and analyze data.

The saline used for dissection was haemolymph-like 3(HL3) [23,27]: (in mmol/L) 70 NaCl, 5 KCl, 20 MgCl_2_, 10 NaHCO_3_, 1 CaCl_2_, 5 trehalose, 115 sucrose, 25 N,N-bis(2-hydroxyethyl)-2-aminoethane sulfonic acid (BES) and pH at 7.2. The CaCl_2_ was varied from 0.5 to 3.0 mM as described in the Results for the various experiments. CaCl_2_ was replaced by BaCl_2_, and NaCl replaced by LiCl or choline chloride in experiments mentioned. Two other buffers besides BES were examined for their suitability. These were Trizma^®^ base (2-amino-2-(hydroxymethyl)-1,3-propanediol) and CAPS (3-(Cyclohexylamino)-1-propanesulfonic acid). All chemical were from Sigma-Aldrich (St. Louis, MO, USA).

### 2.3. Statistical Analysis

Data are expressed as averages (±SEM-standard error of the mean). Response differences before and after solution exchange were quantified with paired *t*-tests. The Shapiro-Wilk test was used to establish normality. When appropriate, the Wilcoxon rank sum, non-parametric test was used. Two-way analysis of variance (ANOVA) was performed with multiple comparisons among different larval strains with Tukey’s method since sample sizes were the same. Sigma Stat software (version number 15.0) was used for analysis, and a *p*-value of *p* < 0.05 was considered statistically significant.

## 3. Results

### 3.1. Altering [Ca^2+^]_O_ Effects on Membrane Potential

**Figure 1:**
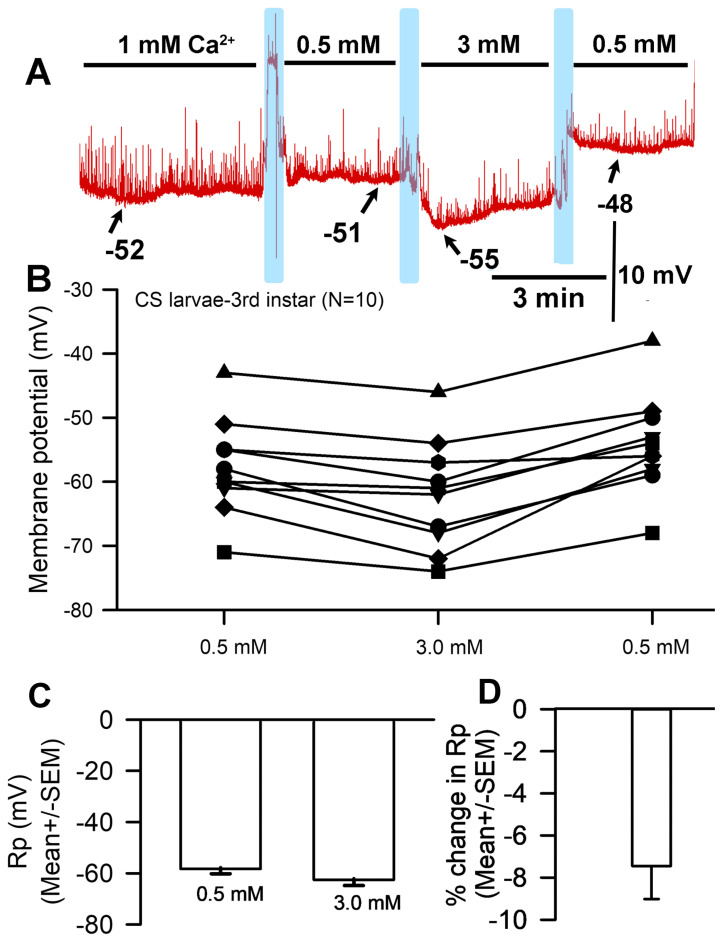
The effects on resting membrane potential of altered extracellular [Ca^2+^]. (**A**) A representative recording of the membrane potential with alterations of the bathing medium. (**B**) The responses for 10 individual preparations as [Ca^2+^]_O_ was changed. Raised [Ca^2+^]_O_ led to significant hyperpolarization of the membrane (paired *t*-test; *p* < 0.05) compared to initial values. (**C**) The average membrane potential for 0.5 mM [Ca^2+^]_O_ and 3 mM [Ca^2+^]_O_ (mean ± SEM) showed no significant difference due to the large variation in membrane potentials among preparations. (**D**) The mean percent change for each of the 10 individual preparations (mean ± SEM). The light blue boxes represent exchange of the medium from one solution to the next.

Membrane potential underwent acute hyperpolarization with increased [Ca^2+^]_O_. Since larval body wall muscle was used, the presynaptic motor nerve terminal continued to exhibit spontaneous vesicular fusion events (i.e., minis or quantal events) illustrated by rapid upward deflections on the recording. The motor nerve was transected from the CNS, resulting in a continued observance of spontaneous events that decreased in frequency as time proceeded. Exposure to 3 mM Ca^2+^ affected the muscle fiber membrane within seconds and the effect was generally maintained throughout the full three minutes of observation (Figure 1A). Hyperpolarization occurred when the bath was exchanged from 0.5 mM to 3 mM (Figure 1B), and this effect was found to be significant (paired *t*-test; *p* < 0.05; *n* = 10) with an average percent change of 7.5 (SEM ± 1.6) mV (Figure 1C,D). Some preparations showed greater changes than others but overall, there was a hyperpolarization with exposure to 3 mM. The rationale behind changing the medium from normal saline (with [Ca^2+^]_O_ at 1 mM) to the low 0.5 mM experimental concentration was that this alteration would increase the difference between the low and high calcium environments and thus accentuate the observed effects on Rp. If [Ca^2+^]_O_ is removed from the saline, there is a higher probability of the motor nerve firing action potentials and causing muscle contraction, which can dislodge the intracellular electrode; thus, 0.5 mM was the lowest concentration used in this paradigm.

### 3.2. Choline Chloride Experiments

**Figure 2:**
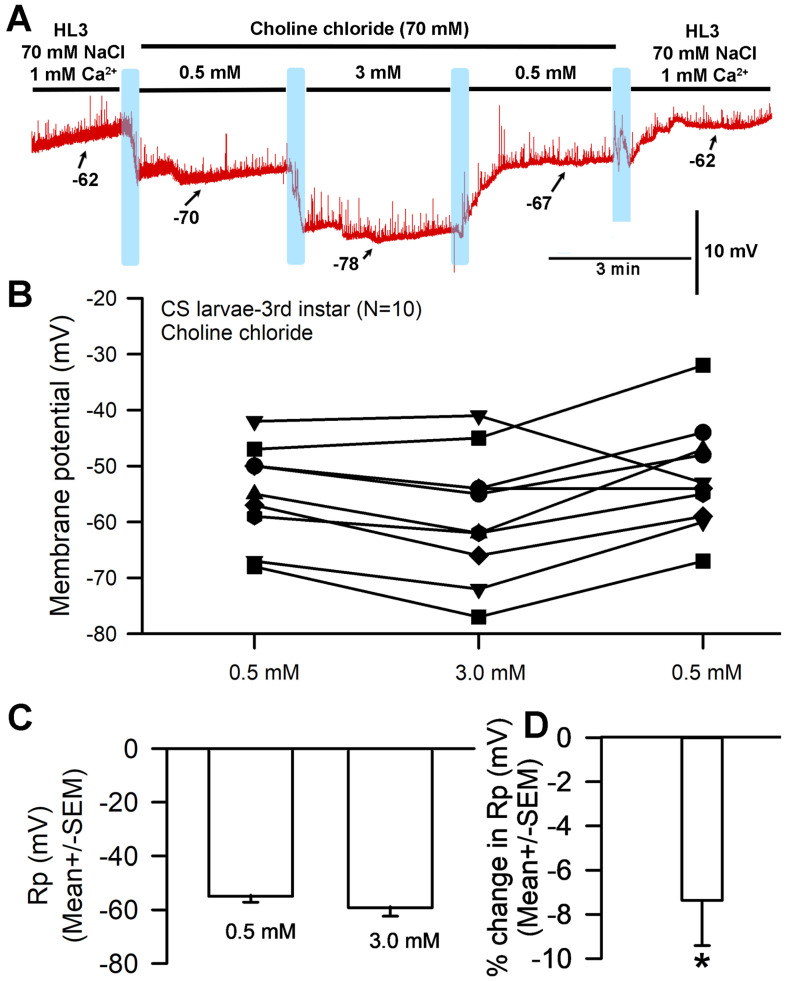
The effects on resting membrane potential of substituting choline chloride for NaCl and altering extracellular [Ca^2+^], but with Na+ present from NaHCO_3_^−^ and the NaOH used for pH adjustments. (**A**) A representative trace of the membrane potential as the medium is changed from saline containing NaCl to that containing choline chloride, to 0.5 mM [Ca^2+^]_O_, to 3.0 mM, back to 0.5 mM, and then to HL3 saline once more. (**B**) The responses for 10 individual preparations during altered [Ca^2+^]_O_ while exposed to choline chloride instead of NaCl. Raised [Ca^2+^]_O_ led to significant hyperpolarization of the membrane (paired *t*-test; *p* < 0.05). (**C**) The average membrane potential for 0.5 mM [Ca^2+^]_O_ and 3 mM [Ca^2+^]_O_ (mean ± SEM) showed no significant difference due to the large variation in membrane potentials among preparations. (**D**) The mean percent change from initial for each of the 10 individual preparations (mean ± SEM). The light blue boxes represent exchange of the medium from one solution to the next. There was a significant difference in the percent change from 0.5 to 3.0 mM (* paired *t*-test; *p* < 0.05).

A representative recording in which saline was exchanged for that containing increased Ca^2+^ and a replacement of Na^+^ with choline chloride is depicted in Figure 2A. With the exchange of NaCl (70 mM) in HL3 saline for choline chloride (70 mM) and of [Ca^2+^]_O_ from 0.5 mM to 3.0 mM, significant hyperpolarization of the membrane was observed (Figure 2A,B; paired *t*-test; *p* < 0.05; N = 10). The average percent change was 7.4 (SEM ± 2.1) mV (Figure 2C,D). The response variation observed appears to be a function of normal within-preparation physiological variation.

**Figure 3:**
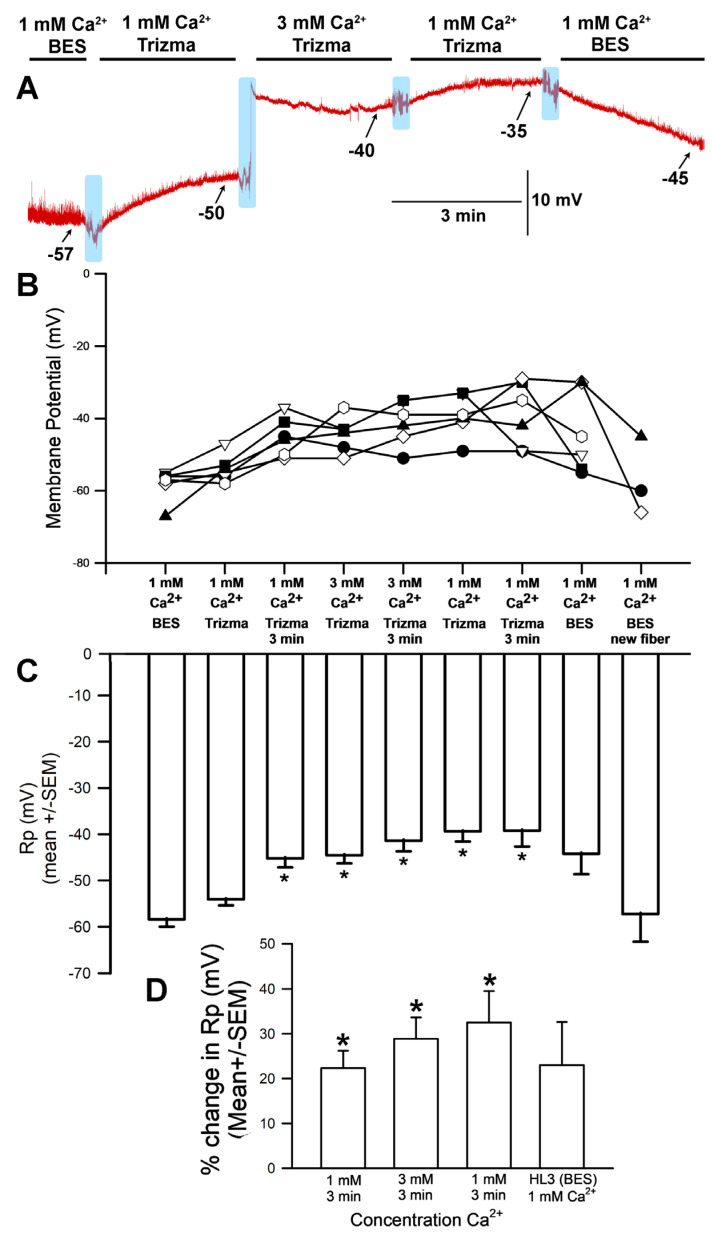
The effects on resting membrane potential of substituting choline chloride for NaCl and Trizma^®^ base for NaHCO_3_, as well as altering [Ca^2+^]_O_. (**A**) A representative trace of the membrane potential as the medium is changed from saline (HL3-BES) containing NaCl to that containing choline chloride/Trizma^®^ base, to 1 mM [Ca^2+^]_O_, to 3.0 mM, back to 1 mM, and then to HL3 saline once more. (**B**) The responses for 10 individual preparations during altered [Ca^2+^]_O_ while exposed to choline chloride and Trizma^®^ base. (**C**) The average membrane potential in HL3 saline, 1 mM [Ca^2+^]_O_, 3 mM [Ca^2+^]_O_, 1 mM [Ca^2+^]_O_ again, and HL3 again (mean ± SEM). Significant depolarization of the membrane was observed (* *p* < 0.05, ANOVA for initial HL3-BES to 1 mM after 3 min of incubation and 3 mM [Ca^2+^]_O_ as well as to returning to 1 mM in Trizma^®^; Paried *t*-test were significant *p* < 0.05 for comparing initial HL3-BES to 1 mM after 3 min of incubation and 3 mM [Ca^2+^]_O_ as well), though it hyperpolarized again when the medium was returned to HL3-BES saline. (**D**) The mean percent change from initial for each of the 10 individual preparations (mean ± SEM). The light blue boxes represent exchange of the medium from one solution to the next. There was a significant difference in the percent changes, * paired *t*-test; *p* < 0.05).

**Figure 4:**
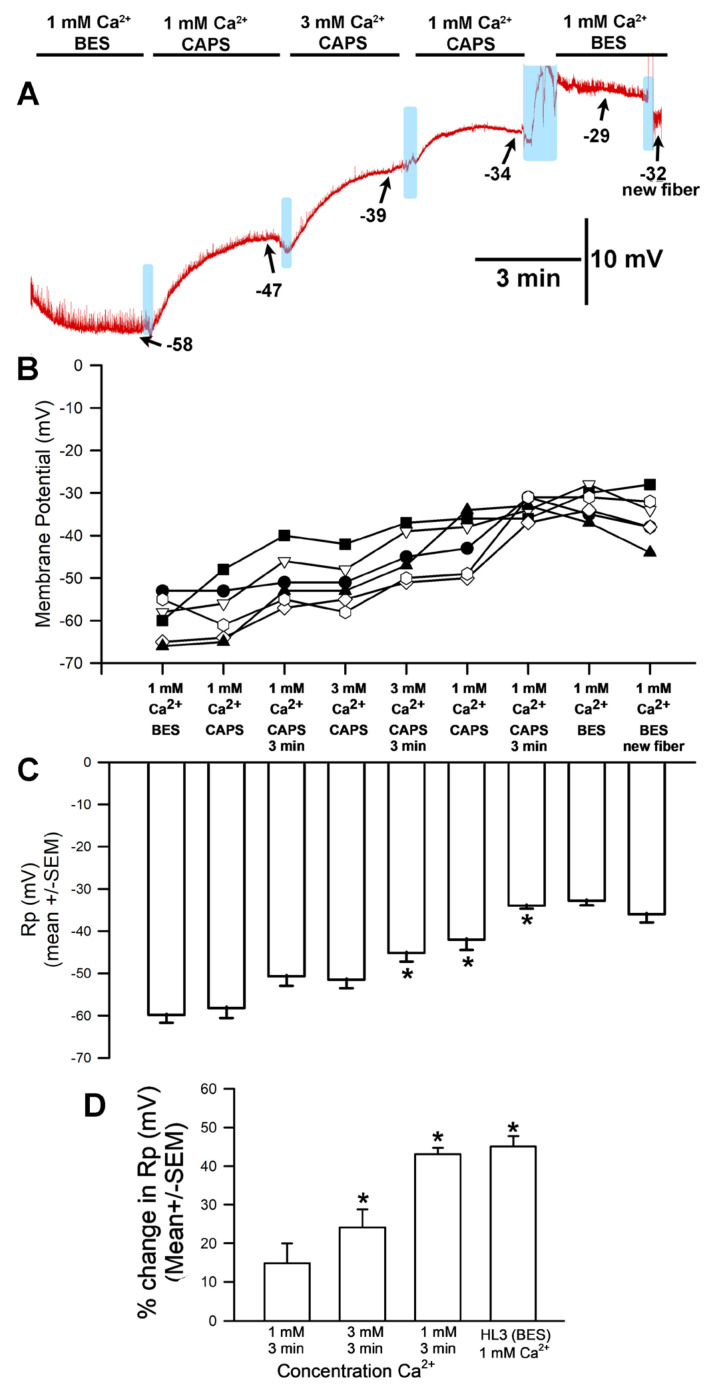
The effects on resting membrane potential of substituting choline chloride for NaCl and CAPS for NaHCO_3_, as well as altering extracellular [Ca^2+^]. (**A**) A representative trace of the membrane potential as the medium is changed from saline containing NaCl to that containing choline chloride/CAPS, to 1 mM [Ca^2+^]_O_, to 3.0 mM, back to 1 mM, and then to HL3 saline once more. (**B**) The responses for 10 individual preparations during altered [Ca^2+^]_O_ while exposed to choline chloride and CAPS. Significant depolarization of the membrane was observed (ANOVA for initial HL3-BES to 3 mM [Ca^2+^]_O_ as well as to returning to 1 mM in CAPS; Paried *t*-test were significant *p* < 0.05 for comparing initial HL3-BES to 1 mM after 3 min of incubation and 3 mM [Ca^2+^]_O_ as well) and did not re-hyperpolarize even upon return to HL3 saline or examination of adjacent muscle fibers. (**C**) The average membrane potential in HL3 saline, 1 mM [Ca^2+^]_O_, 3 mM [Ca^2+^]_O_, 1 mM [Ca^2+^]_O_ again, and HL3 again (mean ± SEM). Significant differences were observed (* *p* < 0.05, ANOVA for percent difference of HL3-BES 3 min to 1 mM CAPS at 3 min to 3 min of 3 mM [Ca^2+^]_O_ as well as to returning to 1 mM in CAPS; * Paired *t*-test were significant *p* < 0.05). (**D**) The mean percent change from initial for each of the 10 individual preparations (mean ± SEM). There was a significant difference in the percent changes, * paired *t*-test; *p* < 0.05. The light blue boxes represent exchange of the medium from one solution to the next.

The hyperpolarization observed after exposure to 3 mM [Ca^2+^]_O_ (with choline chloride in place of NaCl in the saline) was unexpected for a NaCl-free solution; in such a situation, no significant difference in membrane potential would be expected between 1 mM and 3 mM [Ca^2+^]_O_. This response is likely because the HL3 saline used both contained NaHCO_3_ and was brought to a pH of 7.2 using NaOH, which would result in the presence of additional sodium in the solution despite the removal of NaCl. These experiments were thus redone with new solutions that contained choline chloride in place of NaCl while replacing NaHCO_3_ with, respectively, Trizma^®^ base (Figure 3) and CAPS (Figure 4) in an effort to eliminate sodium from the solution. HCl was used to adjust the pH to 7.2.

### 3.3. LiCl Experiments

**Figure 5:**
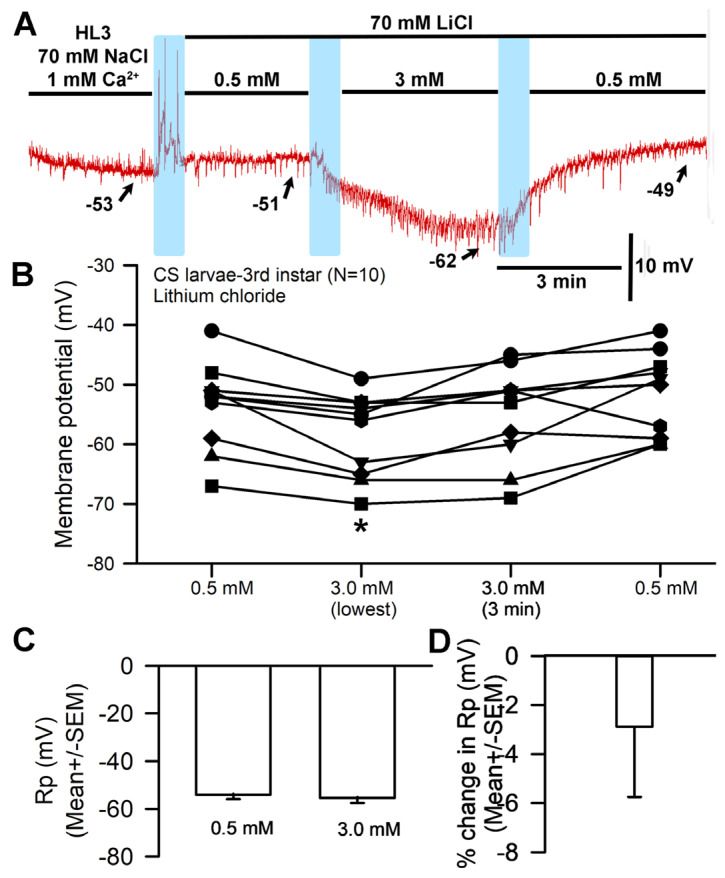
The effects of replacing NaCl with LiCl and altering [Ca^2+^]_O_ on resting membrane potential. (**A**) A representative trace of the membrane potential change observed when NaCl is replaced with LiCl and during subsequent exposure to [Ca^2+^]_O_ 0.5 mM to 3.0 mM and back. (**B**) The responses observed across 10 individual preparations as [Ca^2+^]_O_ is changed during LiCl exposure. The effect of raised [Ca^2+^]_O_ produced significant hyperpolarization of the membrane (paired *t*-test; * *p* < 0.05) compared to initial values. (**C**) The average membrane potential between 0.5 mM [Ca^2+^]_O_ and 3 mM [Ca^2+^]_O_ (mean ± SEM) showed no significant difference among preparations after 3 min. (**D**) The mean percent change for each of the 10 individual preparations (mean ± SEM). The light blue boxes represent exchange of the medium from one solution to the next.

Another paradigm involved replacing the NaCl (70 mM) present in HL3 saline with LiCl at 70 mM. A representative recording is shown in Figure 5A. The hyperpolarization was delayed in the Li^+^ saline for some preparations as compared to HL3 (Figure 1A) and choline-chloride-containing saline (Figure 2A). Observations were conducted over a three-minute period and the largest hyperpolarization within that time was used. In some cases, the membrane potential depolarized from its most hyperpolarized state by the end of the three minutes (Figure 5B,C). The saline containing Li^+^ also demonstrated a significant hyperpolarization when the bath was exchanged from one containing 0.5 mM Ca^2+^ to 3.0 mM Ca^2+^ (paired *t*-test; *p* < 0.05; N = 10). The percent change from 0.5 mM to the end of 3 min’ incubation in 3.0 mM Ca^2+^ saline solution resulted in a hyperpolarization of 2.9 (SEM ± 2.8) mV (Figure 5D).

### 3.4. BaCl_2_ Experiments

**Figure 6:**
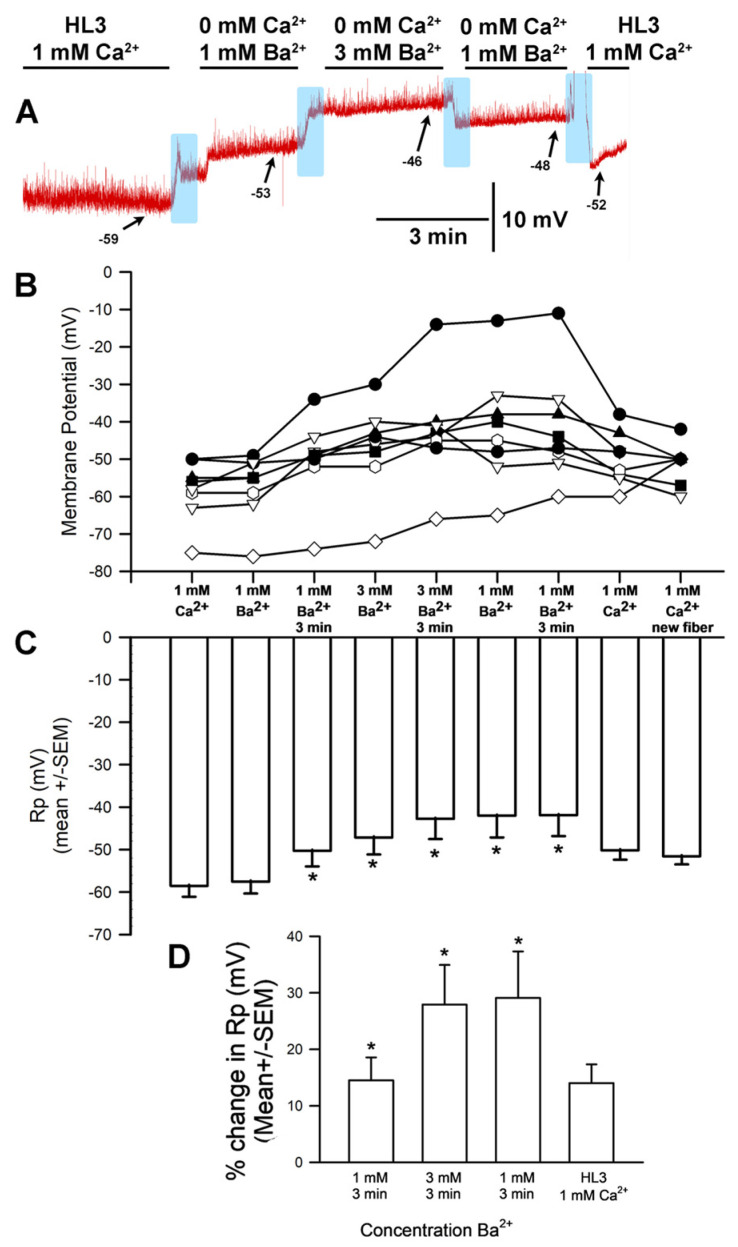
The effects on resting membrane potential of substituting BaCl_2_ for CaCl_2_ and altering [Ba^2+^]_O_. (**A**) A representative trace of the membrane potential as the medium is changed from saline containing CaCl_2_ to that containing 1 mM BaCl_2_, to 3.0 mM BaCl_2_, back to 1 mM BaCl_2_, and then to HL3 saline once more. (**B**) The responses for 10 individual preparations during replacement of CaCl_2_ with 1 mM BaCl_2_, then 3 mM BaCl_2_, back to 1 mM BaCl_2_, and then back to HL3. (**C**) The average membrane potential for 1 mM [Ba^2+^]_O_ and 3 mM [Ba^2+^]_O_ (mean ± SEM) was significantly different (Not normally distributed, thus used a Wilcoxon Signed Rank Test, *p* = 0.031) (**D**) The mean percent change from initial for each of the 10 individual preparations (mean ± SEM). Raised [Ba^2+^]_O_ led to significant depolarization of the membrane potential for a percent change, * paired *t*-test; *p* < 0.05). The light blue boxes represent exchange of the medium from one solution to the next.

To examine whether the hyperpolarizing effects of increased [Ca^2+^]_O_ was specific to the Ca^2+^ ion, another divalent ion was investigated. Ba^2+^ can generally replace Ca^2+^ flux through Ca^2+^ channels, so the CaCl_2_ normally present in HL3 saline was replaced with BaCl_2_. The general trends are shown for a representative preparation (Figure 6A). Replacing HL3 saline containing 1.0 mM CaCl_2_ with that containing 1 mM BaCl_2_ resulted in a significant depolarization of the membrane (*p* < 0.05; paired t-test, N = 10). Likewise, raising [Ba^2+^]_O_ from 1 to 3 mM led to significant depolarization of the membrane (paired *t*-test; *p* < 0.05). The average membrane potential for 1 mM [Ba^2+^]_O_ and 3 mM [Ba^2+^]_O_ (mean ± SEM) was significantly different (Figure 6B,C; The values were not normally distributed; thus, a Wilcoxon Signed Rank Test was used, *p* = 0.031). The average percent change from HL3 with 1 mM CaCl_2_ to 1 mM BaCl_2_ (7.2 SEM ± 3 mV) and from 1 mM BaCl_2_ to 3.0 mM BaCl_2_ (6.1 SEM ± 2.1 mV) are shown in Figure 6D. The percent change was taken from the end of the three minutes’ exposure in one solution to the end of three minutes’ exposure in the next.

### 3.5. The Effects on Membrane Potential in Muscle Overexpressing K2P Channels

**Figure 7:**
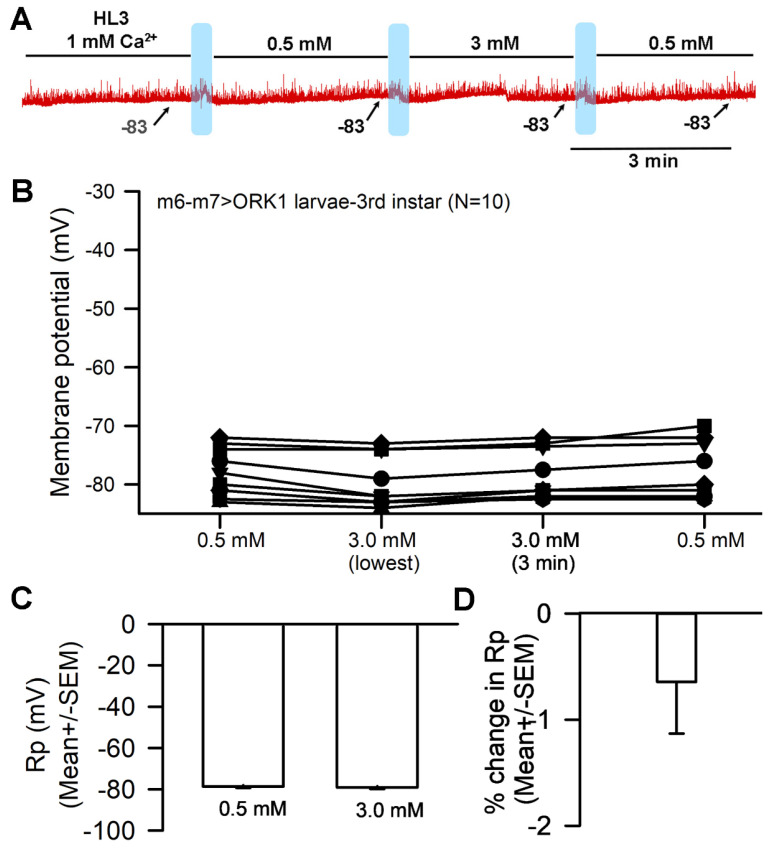
The effects of altering [Ca^2+^]_O_ on resting membrane potential for muscle overexpressing K2P channels. (**A**) A representative trace of the membrane potential changes as [Ca^2+^]_O_ is changed from 1.0 mM to 0.5 mM to 3.0 mM and back to 0.5 mM. (**B**) The responses for 10 individual preparations as [Ca^2+^]_O_ were changed. The effect of raising [Ca^2+^]_O_ from 0.5 to 3.0 mM had no significant hyperpolarization effect on the membrane (paired *t*-test; *p* > 0.05). (**C**) The average membrane potential for 0.5 mM [Ca^2+^]_O_ and 3 mM [Ca^2+^]_O_ (mean ± SEM) showed no significant difference among preparations. (**D**) The mean percent change for each of the 10 individual preparations (mean ± SEM). The light blue boxes represent where the exchange of saline took place.

The genetically modified *Drosophila* line (m6-m7 > ORK1, bearing an overexpression of the ORK1 K2P channel subtype) underwent no significant effects to membrane potential when exposed to raised [Ca^2+^]_O_. The response for a representative preparation is shown (Figure 7A). The muscle fiber membrane potential was not significantly altered by the exchange from 0.5 to 3 mM Ca^2+^ (Figure 7B,C) (*p* > 0.05; Paired *t*-test; N = 10), and the average percentage change was also minor, with no significant effect (Figure 7D).

## 4. Discussion

In this investigation, it was demonstrated that membrane potential was affected by [Ca^2+^]_O_ such that raising the concentration resulted in hyperpolarization, and vice versa. Replacing the NaCl in the saline with LiCl or choline chloride and then exchanging the CaCl_2_ from 0.5 mM to 3.0 mM caused a hyperpolarization of the membrane. Replacing CaCl_2_ with BaCl_2_ led to depolarization of the membrane and increasing the BaCl_2_ from 0.5 mM to 3.0 mM resulted in further depolarization. In larval muscle overexpressing K2P channels (ORK1), the effects of increased [Ca^2+^]_O_ on membrane potential were greatly reduced, likely because high K^+^ permeability with more leak channels causes a strong tendency to remain at E_K_; this indicates that the ion channel density and K2P channel subtypes present change how membrane potential is affected by altered [Ca^2+^]_O_. The results herein confirm previous findings that larval *Drosophila* muscle hyperpolarizes with raised [Ca^2+^]_O_ [4]. 

To investigate potential mechanisms, Ca^2+^ was replaced with Ba^2+^, which resulted in depolarization instead of hyperpolarization. Given that the resting membrane resides not at E_K_ but a slightly depolarized value, the data suggest an ionic leak, such as a small Na^+^ leak. Considering the possibility that Ca^2+^ ions may block NALCN channels, as has been suggested for other cell types [8,9,11,12], increasing [Ca^2+^]_O_ would potentially block more NALCN channels, resulting in a greater drive towards E_K_. If Ba^2+^ displaced any residual Ca^2+^ ions from the channels, however, and thus reduced the blockage of NALCN channels, it would likely result in depolarization. If Ba^2+^ leaked through a NALCN channel, this would also cause depolarization, but there is no precedent to indicate this.

Experimental paradigms addressing the effects of altered [Ca^2+^]_O_ on synaptic efficiency should consider this phenomenon, as one might otherwise focus only on the presynaptic influence of increased Ca^2+^ flux via more evoked vesicle fusion events and greater post-synaptic amplitudes. However, alterations in the Ca^2+^ concentration gradient would also affect the electrical driving gradient, such that elevated [Ca^2+^]_O_ might hyperpolarize the neuronal membrane potential and lead to removal of the inactivation from some voltage-gated Na^+^ channels present, resulting in a lowered excitation threshold for the neuron. Hyperpolarization of the target cell could also influence ionic driving gradients and action potential threshold in the same manner as for the presynaptic neuron. Finally, the membrane potential of larval *Drosophila* muscle is also pH-sensitive [28]; this may impact some K2P channel subtypes, given that the TASK subtype is blocked by acidic conditions [29], and may also affect Ca^2+^ interaction with NALCN channels. Thus far, this topic has largely gone unaddressed, experimentally. It is also of interest to investigate whether the ionized [Ca^2+^] may be decreased by the sodium bicarbonate buffer used in saline or by interactions with other compounds.

The concentration of free Ca^2+^ ions is regulated by various means in intact organisms (hormones, calcium binding proteins) as well as within cells and cellular organelles. The physiological range in human serum is considered to be 2.25 to 2.75 mmol/L for neonates and adults [30,31], while the cerebrospinal fluid (CSF) in healthy humans contains ionized (~1 mM) and total (~1.2 mM) calcium concentration in the CSF to match concentrations in the brain extracellular fluid [32,33]. A major buffer of ionized Ca^2+^ in CSF is carbonate. As is the case for albumin, Ca^2+^ is much lower in CSF than in serum, so regulating saline pH may alter the assumed concentration of ionized Ca^2+^ depending on the buffer used to control pH. Given that chemical synaptic transmission is strongly influenced by Ca^2+^ entry to the presynaptic terminal, the mechanisms behind this may not entirely be due to a concentration gradient, as both the electrical gradient and the modulation of ion channels by the electrostatic interactions can have a role.

Despite years of research with genomic and proteomic tools, it is not yet known how many subtypes of K2P and NALCN channels are expressed and functional within any single cell in which the membrane potential can be measured. In time, it will be possible to know which of the 11 genes known to express K2P channels in *Drosophila melanogaster* are expressed in various cell types across development; in the meantime, however, pharmacological and environmental changes can aid in investigating some of the subtypes present in the membranes. Cold temperatures, acidic conditions, and the compound doxapram (an inhibitor of a K2P-TASK subtype) are known to depolarize larval *Drosophila* muscle [28,34,35]. However, with a pH of 5 or a high concentration of doxapram, the resting membrane does not rapidly reach a potential of zero or E_Na_, suggesting that many other factors (such as pumps, exchangers, and different K2P channel subtypes) may be at play in maintaining a membrane potential. On the other hand, overexpressing a dORKA1 K2P channel in larval *Drosophila* muscle produces membrane potentials that are much more negative and closer to E_K_. [26]. With more leak channels present and, thus, a higher K^+^ permeability, it would be surprising to observe altered [Ca^2+^]_O_ (and the resulting reduction or enhancement of Na^+^ leak channel blockage) affecting membrane potential. Rather, as demonstrated in this study, the membrane potential of a given cell would vary depending on the density of K^+^ and Na^+^ leak channels present.

The NALCN channel subtype expressed in the skeletal muscle of larval *Drosophila* has yet to be identified; however, it is likely that a form of Na^+^ leak channel is present, as the Rp is not maintained at Ek (~−90 mV) and overexpression of a K2P channel hyperpolarizes the membrane potential from wild-type *Drosophila*. An NALCN antibody for a mammalian channel subtype is commercially available [2], and it might be worthwhile to accumulate *Drosophila* body wall tissue and test the antibody’s effectiveness in the model through the use of Western blots. Some pharmacological agents block NALCN channels in mammalian tissues and could potentially affect *Drosophila* preparations as well [2]. It would also be of interest to use RNA-Seq to examine the effects of altered K2P and NALCN channel expression in other *Drosophila* tissues; this approach would allow RNAi targeting of specific subtypes and tissues, as well as addressing the fact that K2P and NALCN channels are not solely responsible for functional expression because the associated accessory proteins are significant as well. It is even possible that K2P overexpression somehow affected ion channel density or incorporation into the membrane which would not be determined by Western blot or even RNA-Seq. However, these approaches are beyond the scope of this current study. The NALCN protein is associated with various auxiliary subunits (UNC80 and UNC79, as well as others), which form a complex now known as an NALCN channelosome [36]. Examining how these subunits impact channel function could be addressed in future studies with *Drosophila*, as these other proteins are well-established in this model.

Removing all Na^+^ from the bathing medium was problematic because it prevented both pH adjustment with NaOH and use of the NaHCO_3_^−^ buffer. Basic buffers such as CAPS and Trizma^®^ Base were explored as possible replacements to allow pH adjustment with HCl; however, the larval muscle did not fare well with these buffers, as the membrane would depolarize even with [Ca^2+^]_O_ held constant. The molecular structures of CAPS and Trizma^®^ Base differ from the BES buffer, which, as has been previously discovered, best maintains physiological function of the larval heart for the hours needed to conduct in situ studies [27,37]. In the same experiments, a wide array of possible buffers was examined, and none performed so well as BES. It is unknown why CAPS and Trizma^®^ Base altered the membrane potential in this investigation, as pH was maintained at 7.2 and the other salts remained consistent (save for the aforedescribed absence of NaHCO_3_). Other buffers, such as 4-(2-Hydroxyethyl)piperazine-1-ethanesulfonicacid (HEPES), might be feasible replacements, but attempting to use HEPES resulted in an acidic medium that could not be adjusted without using NaOH. Cesium hydroxide (CsOH) could feasibly be used for pH adjustments, but the effects of Cs^+^ in solution would also need to be examined. Other buffers might be useful as replacements, but their effects on cell viability would need to be tested first.

**Figure 8:**
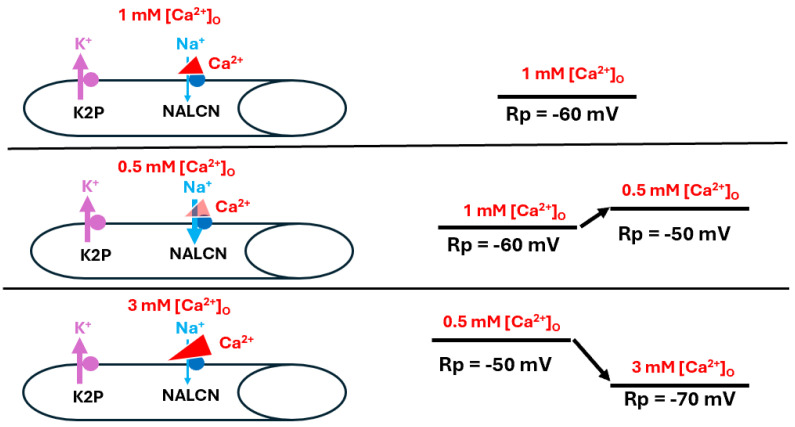
The effects of altered [Ca^2+^]_O_ on resting membrane potential. Ca^2+^ is believed to influence resting membrane potential (Rp) by reducing Na^+^ influx through the corresponding leak channel (NALCN). At rest, when [Ca^2+^]_O_ is at physiological levels, the Na^+^ leak into the muscle is slightly hindered (**top panel**). As [Ca^2+^]_O_ is reduced, the Na^+^ leak is enhanced and Rp depolarizes (**middle panel**). As [Ca^2+^]_O_ is raised, NALCN channels are blocked to a greater degree than observed at physiological [Ca^2+^]_O_ and the Rp is hyperpolarized as it approaches equilibrium potential for K^+^ (**bottom panel**). The thickness of the arrows illustrates the changes occurring.

Various schematic models based on the manipulations used in this study are presented to help explain the overall mechanisms behind the alterations in membrane potential observed here. This first model highlights the effects of raised [Ca^2+^]_O_ on the cells’ membrane potentials (Figure 8).

**Figure 9:**
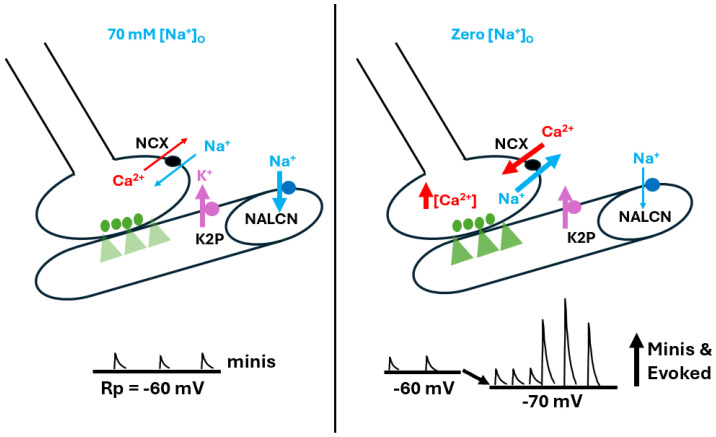
The effects of altered [Na^+^]_O_ on resting membrane potential and spontaneous nerve activity. [Na^+^]_O_ was reduced by replacing the NaCl in physiological saline with equal concentrations of choline chloride (i.e., 70 mM for *Drosophila* saline). Lower [Na^+^]_O_ produced hyperpolarization of the larval muscle’s resting membrane potential, likely due to reduced drive on the Na^+^ leak channel (NALCN). In addition, spontaneous quantal events occurred, as well as large postsynaptic excitatory junction potentials (EJPs) appearing as nerve-evoked EJPs. It is apparent that more single and multi-quantal events occurred, which indicates a presynaptic response that may be driven by the passive sodium-calcium exchanger (NCX). This response likely results in an influx of Ca^2+^ due to the low driving gradient for Na^+^ influx, particularly compared to the stronger gradient for Na^+^ efflux. In addition, Na^+^ might even efflux from the muscle fiber. The thickness of the arrows illustrates the changes occurring.

The second model illustrates the effects of reducing Na^+^ through replacement with choline chloride to maintain osmolarity while reducing [Na^+^]_O_ (Figure 9).

**Figure 10:**
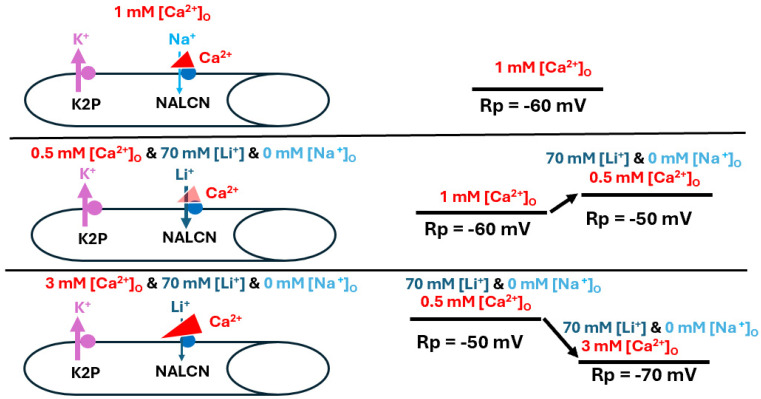
Alterations in [Ca^2+^]_O_ during substitution of Li^+^ for Na^+^ affect resting membrane potential. In basal conditions Ca^2+^ is blocking some of the NALCN channels (**top panel**). Alterations in [Ca^2+^]_O_ after replacement of Na^+^ with Li^+^ resulted in similar responses to those observed when Na^+^ was present. Reducing [Ca^2+^]_O_ likely decreased the slight blockage of the Na^+^ leak channel (NALCN), resulting in depolarization of the muscle fiber (**middle panel**). On the other hand, raising [Ca^2+^]_O_ enhanced the block, promoting the action of K2P channels and resulting in membrane hyperpolarization towards the equilibrium potential for K^+^ (**bottom panel**). The thickness of the arrows illustrates the changes occurring.

The third model illustrates the replacement of NaCl with LiCl and subsequent alterations of [Ca^2+^]_O_, which indicates that raised [Ca^2+^]_O_ results in more blockage of the Na^+^ leak (Figure 10). The explanation behind increased [Ca^2+^]_O_ hyperpolarizing the membrane in this paradigm is not fully understood. It is possible that the sodium-potassium pump or other pumps/exchangers work differently in these conditions [38].

**Figure 11:**
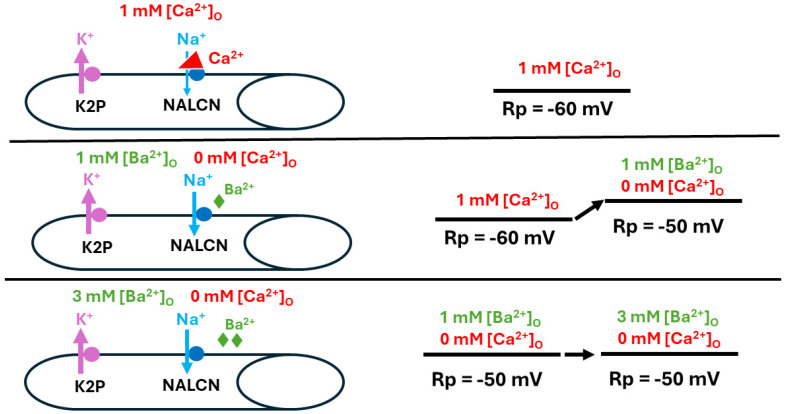
Replacing Ca^2+^ with Ba^2+^ and its effect on resting membrane potential. When [Ca^2+^]_O_ is at physiological levels, the Na^+^ leak (NALCN) channels in the muscle is slightly blocked (**top panel**). After removing Ca^2+^ and replacing it with Ba^2+^ at the same concentrations used for examining the effects of [Ca^2+^]_O_ alteration on resting membrane potential (Rp) (**middle panel**), increasing [Ba^2+^]_O_ resulted in reduced blockage of NALCN channels (**bottom panel**).

The results upon substituting BaCl_2_ for CaCl_2_ are interesting, as it was expected that the cellular response would be similar to those observed with varying [Ca^2+^]_O_, since Ba^2+^ could potentially block the Na^+^ leak channel in a similar manner. Instead, it appears that the cells slightly depolarized in the BaCl_2_ solution, with greater depolarization observed at higher concentrations (Figure 11). The NALCN channels may be maximally disinhibited even at 0.5 mM Ba, such that no potential change would be expected upon raising Ba^2+^ to 3 mM (Figure 11, bottom panel).

BaCl_2_ has been used in past studies to address the possibility that calcium influx activates K_(Ca)_ channels from the cytoplasmic side or through direct blockage of voltage-gated K^+^ channels [14,39,40]. It appears as though replacing Ca^2+^ with Ba^2+^ enables Ba^2+^ influx through Ca^2+^ channels without activation of K_(Ca)_ channels. In the muscle cells studied during this investigation, it is not likely that the hyperpolarization stems from a Ca^2+^ leak activating K_(Ca)_ channels, as such a leak would likely result in muscle contractions not observed in this study. Spontaneous quantal events weren’t observed either, which would also be expected in the presence of increased [Ca^2+^]_O_. However, Ba^2+^ might block the K2P channel, as is known to occur for voltage-gated K^+^ channels [3].

**Figure 12:**
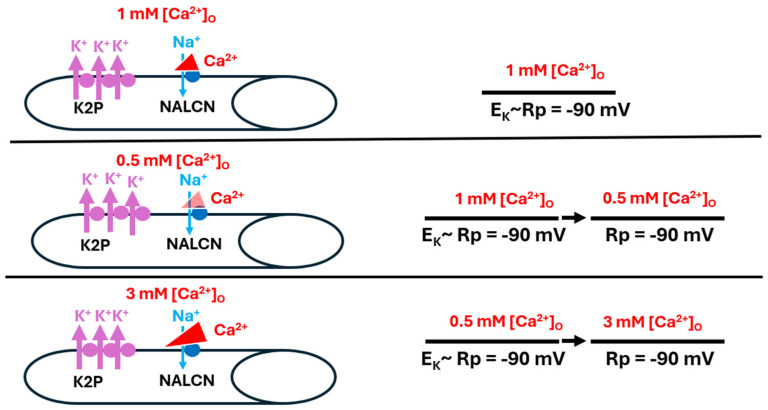
When K2P channels are overexpressed in muscle fibers, resting membrane potential (R_p_) is not influenced by altered [Ca^2+^]_O_. Rp was observed with Ca^2+^ reducing Na^+^ influx through the Na^+^ leak channel (NALCN) at 1 mM (**top panel**), with a reduced concentration of 0.5 mM [Ca^2+^]_O_ (**middle panel**), and with a raised concentration of 3 mM [Ca^2+^]_O_ (**bottom panel**). In each condition, the permeability of Na^+^ (P_Na_) was negligible as compared to the high permeability of K^+^ (P_K_), driving Rp towards the equilibrium potential for K^+^ (E_K_) (**bottom panel**).

Cells would likely exhibit very negative resting membrane potentials if they also demonstrated a reduced level of Na^+^ leak or a higher expression of functional K2P channels; the results thus indicate a high level of K^+^ leak that renders negligible the effects from both the Na^+^ leak and the Ca^2+^-induced blockage of the Na^+^ leak (Figure 12).

It is not likely that K_(Ca)_-channel activation accounts for the hyperpolarization observed with raised [Ca^2+^]_O_. The muscle does not appear to respond well to elevated [Ca^2+^]_O_, as the muscle shows granulation over time; it appears as though, over the 10 min of observation, increased [Ca^2+^]_O_ damages the muscle cell in CS strains but not in K2P-overexpressing strains. Ca^2+^ ions were noted to block voltage-gated ion channels and have an influence on the kinetics of various channels [8]. While recording compound action potentials (CAPs) in marine crab nerves, it was demonstrated that replacing CaCl_2_ with BaCl_2_ resulted in depressed CAP amplitude, possibly indicating a blockage of some voltage-gated Na^+^ channels [5]. Earlier studies addressed the potential that Ca^2+^ screens charges on membranes [13]. One might have expected BaCl_2_ to affect membrane potential similarly to CaCl_2_.

In the future, it would be of interest to investigate the distribution of ion channel subtypes responsible for the resting membrane potential; it would also be worthwhile to model ion permeabilities and concentration differences through simulations of the Goldman-Hodkin-Katz equation, which would allow examination of how slight differences in leak channel expression (both K2P and NALCN) affect organisms. Since the expression of K2P channels in human tissues varies under pathological conditions, such as cancer [15,16,29], it would also be of interest to investigate the impact of varied ionic concentrations on different cell types with varied membrane potentials.

## 5. Conclusions

Changing external calcium concentrations from 0.5 to 3 mM led to hyperpolarization of the muscle. Replacing NaCl with LiCl or choline chloride still led to hyperpolarization as the calcium concentration increased. Replacing CaCl_2_ with BaCl_2_ results in depolarization. Larval muscle bearing K2P channel overexpression largely saw greatly reduced effects with altered [Ca^2+^]_O_, likely because potential is heavily driven by the E_K_ in these muscles.

## Data Availability

The data presented in this study are available on request from the corresponding author. The data are not publicly available due to requiring specific software to view the data files. Most of the data are presented within this publication in the line graphs.
